# High-NA two-photon single cell imaging with remote focusing using a diffractive tunable lens

**DOI:** 10.1364/BOE.405863

**Published:** 2020-11-16

**Authors:** Molly A. May, Martin Bawart, Michiel Langeslag, Stefan Bernet, Michaela Kress, Monika Ritsch-Marte, Alexander Jesacher

**Affiliations:** 1 Institute of Biomedical Physics, Medical University of Innsbruck, Müllerstraße 44, 6020 Innsbruck, Austria; 2 Institute of Physiology, Medical University of Innsbruck, Schöpfstraße 41, 6020 Innsbruck, Austria

## Abstract

Fast, volumetric structural and functional imaging of cellular and sub-cellular dynamics inside the living brain is one of the most desired capabilities in the neurosciences, but still faces serious challenges. Specifically, while few solutions for rapid 3D scanning exist, it is generally much easier to facilitate fast in-plane scanning than it is to scan axially at high speeds. Remote focusing in which the imaging plane is shifted along the optical axis by a tunable lens while maintaining the position of the sample and objective is a promising approach to increase the axial scan speed, but existing techniques often introduce severe optical aberrations in high-NA imaging systems, eliminating the possibility of diffraction-limited single-cell imaging. Here, we demonstrate near diffraction-limited, volumetric two-photon fluorescence microscopy in which we resolve the deep sub-micron structures of single microglia cells with axial scanning performed using a novel high-NA remote focusing method. Image contrast is maintained to within 7% compared to mechanical sample stepping and the focal volume remains nearly diffraction-limited over an axial range greater than 86 µm.

## 1. Introduction

A broad range of clinical and fundamental questions in the biological sciences could be addressed with fast, volumetric optical microscopy at the single cell level, but functional cellular imaging over large volumes on biologically relevant time scales remains challenging. This is in large part due to the significant time required for axial translation of the focal plane [1–3]. For example, mapping the activity of neuronal networks deep inside the living brain is one of the most pressing problems in neuroscience, and while innovative solutions like two-photon microscopy allow high resolution imaging through thick layers of scattering tissue, and resonant scan mirrors or acousto-optic deflectors allow in-plane scan frequencies of several kHz, three dimensional imaging with spatial resolution on the scale of individual dendrites and temporal resolution on the scale of single action potentials is still limited by a lack of solutions for fast axial scanning. Namely, mechanical translation of the objective or sample limits scan speeds to a few 100 Hz at best, but this introduces vibrations to the sample and results in a nonlinear objective movement demanding calibrational steps [4].

Remote focusing has attracted significant attention as a fast, non-invasive alternative to mechanical axial scanning in which the focal plane of an imaging system is shifted along the optical axis by an auxiliary focusing element while maintaining the position of the sample and objective lens. Many approaches to remote focusing exist [5–23], several of which allow for axial scan speeds up to three orders of magnitude faster than mechanical translation, thus opening the door to volumetric imaging of cellular dynamics on sub-ms timescales. But, most approaches to rapid remote focusing introduce strong optical aberrations to the focal spot, especially when used with high-NA imaging systems, which makes them unsuitable for high resolution single cell microscopy [10,12,15,24,25]. Furthermore, many techniques require complex or expensive hardware and are not easily integratable into the optical path of a standard microscope [20,21,26–28].

Here, we employ a recently developed remote focusing technique based on the Alvarez-Lohmann tunable lens principle [29,30] in which two stacked Moiré diffractive optical elements (MDOE) [31,32] impart a high-NA compatible defocus phase function on the excitation light, leading to an axial shift of the focal plane that increases linearly with the relative rotation between the elements as described in [33]. Because translation of the focal plane requires only the rotation of an MDOE element, this design allows for continuous axial scanning with minimal acceleration of masses, theoretically enabling kHz scan speeds with existing motorized rotation mounts.

With this novel MDOE lens design, we demonstrate near diffraction-limited, high-NA two-photon fluorescence imaging of microglia cells in GFP stained spinal tissues with axial scanning by remote focusing. Furthermore, we demonstrate that this method causes no measurable degradation in imaging resolution compared to optical sectioning by mechanical z-stepping of the sample and only a minimal decrease in the image contrast. A quantitative investigation of the two-photon imaging resolution is performed by measuring the 2-photon point spread function (PSF), revealing that aberrations associated with the MDOE cause just a slight elongation of the focal volume, even for rotation angles up to $\pm 120^{\circ }$, corresponding to translation of the focal plane over a range of 86.6 μm. For comparison, the maximum PSF stretch caused by the MDOE is nearly an order of magnitude less than that introduced by replacing the MDOE with an achromatic lens doublet that shifts the focus position by 50 µm.

This demonstration of robust, practically aberration-free remote focusing in high resolution nonlinear cellular imaging opens the door to rapid, volumetric sub-cellular biological microscopy at increased scan speeds.

## 2. Experiment

Remote focusing was performed using a rotational diffractive Alvarez lens in which a pair of Moiré diffractive optical elements (Diffratec MI-10-1064) produce a spherical defocus phase function Φ(r) given by

(1) $$\Phi (r,\theta )=k_{n}\sqrt{1-{\left(\frac{r}{n\;f}\right)}^{2}}\cdot \Delta z_{0}\cdot \theta$$

where $k_{n}$ is the wave vector in a medium with refractive index n, f is the focal length of the objective lens, r is the radial pupil coordinate, $\Delta z_{0}$ is the strength of the MDOE with units of focal shift per radian of rotation θ between the two elements [33]. Importantly, this phase function was recently shown to maintain a near diffraction-limited focal spot even for high-NA imaging systems, which is a significant improvement over previous designs and essential for imaging complex structures [33]. Additionally, the phase function is rounded $\Phi_{RND}(r)=round(\Phi (r))$ to avoid azimuthal phase discontinuities which lead to the formation of multiple foci or *sectors*. This provides the advantages of maintaining the focal shape independent of the relative lens rotation θ and improving the efficiency of the lens at small θ from a linear fall-off with increasing θ to a $sinc{\left(\theta /2\right)}^{2}$ scaling as described in [31]. Note that as the lens efficiency decreases with increasing rotation angle, most of the power goes into a second focus which lies on the same optical axis as the main focus but has a high axial separation of about 120 µm at an excitation wavelength of 800 nm [33]. Hovewer, this extraneous light causes only a minor signal contribution due to the nonlinearity of the two photon response. It thus caused no measurable signal from out of plane structures. The second, undesired focus could be further suppressed by using an aperture placed in an image-conjugate plane after the MDOE (see Supplementary Materials for details).

**Fig. 1.**
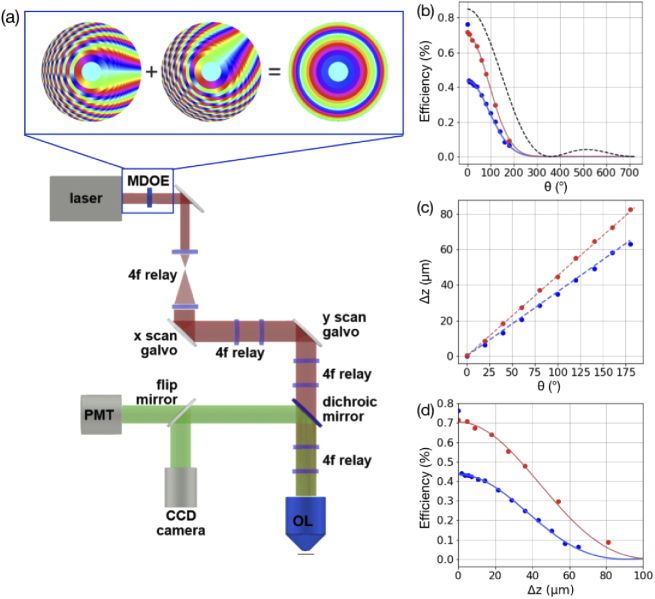
**Experimental layout and lens characterization.** (a) Two-photon scanning microscopy experiment, with MDOE and galvo mirrors imaged onto the back focal plane of the objective using 4f-relays and MDOE lens doublets producing a tunable spherical phase mask shown in the inset. (b) First order MDOE diffraction efficiency as a function of lens rotation for 800 nm (blue) and 1000 nm (red) excitation with fits to the data (solid lines) and theoretical efficiency (black, dashed), deduced from two photon measurements. (c) Focal shift as a function of lens rotation for 800 nm (blue) and 1000 nm (red) excitation and theoretical predictions (dashed lines). (d) First order MDOE diffraction efficiency as a function of focal shift for 800 nm (blue) and 1000 nm (red) with fits to the data shown as solid lines.

A home-built, scanning two-photon excitation microscope (TPEF) with tunable femtosecond laser excitation (Spectra Physics Mai Tai DeepSee) was used for nonlinear fluorescence imaging as shown in Fig. 1(a). The excitation beam was expanded to fill the high-NA MDOE, which was imaged onto the entrance pupil of a water immersion objective lens (OL, Olympus XLUMPLFLN20XW, NA = 1) and the xy-scan galvos (Thorlabs GVS011) using 4f relays. The MDOE was carefully aligned to the OL pupil in the axial direction as well as in the xy-plane, because an offset of the spherical phase function from the pupil can degrade the resulting PSF quality. A dichroic mirror was then used to direct the excitation light through the objective to the sample plane and subsequently to send the filtered fluorescence signal to the detection path where it was measured using either a PMT (Hamamatsu H10682-210) or a CCD camera for diagnostic widefield imaging.

Measurements of efficiency, focal shift, and point spread functions (PSF) were made using the two-photon fluorescence signal from single quantum dots (Thermo Fisher Scientific Qdot 605) dispersed on a glass cover slip, and a piezo stage (Physik Instrumente P-528) was used for precise z-positioning of the sample along the optical axis. During efficiency and focal shift measurements, the two-photon signal was maximized for each MDOE rotation angle using the piezo z-stage and the signal intensity and axial sample position were recorded at this position. It is also worth noting that the two-photon signal intensity scales with the square of the lens efficiency as $sinc{\left(\theta /2\right)}^{4}$, so the data were fit to this function and the square root of the measured efficiency curves and fits were taken to find the linear lens efficiency. PSF measurements were made by stepping a single quantum dot through the focal plane in z-increments of 0.1 µm and recording two-photon images at each axial position with excitation wavelength $\lambda_{ex}$=800 nm. These 3D image stacks were then summed along the y-axis to generate a two dimensional projection of the PSF onto the xz-plane.

Biological imaging was performed on resident, resting CX3CR1-GFP microglia in 300 µm thick transverse sections of PFA fixed spinal dorsal horn tissue from reporter mice (see Supplementary Materials for details). The excitation laser was tuned to $\lambda_{ex}$=900 nm and an additional chromatic filter (BrightLine Basic 525/39) was used to reject endogenous fluorescence from the surrounding tissue. Reference images of the microglia at each depth were taken by translation of the sample along the optical axis using the piezo stage.

## 3. Results

### 3.1 Lens characterization

Figure 1(b) shows measured efficiencies of the first-order focus of the MDOE lens with $\lambda_{ex}$ centered around 800 nm (blue) and 1000 nm (red). The theoretical efficiency (black, dashed line) is also shown, with maximum value η=0.85 chosen to account for reflection losses from the uncoated MDOE elements and a ${\mathrm{sinc}}^{2}$ functional form as discussed above. The data fall off somewhat faster than theoretically predicted, and can be fit nearly perfectly by the functional form $\eta \;sinc{\left(\theta /360^{\circ }\right)}^{2}e^{-\theta^{2}/\delta^{2}}$, where η and δ are free parameters. The exponential decay with $\delta =177^{\circ }$ accounts for additional angle-dependent losses, at least some of which are associated with axial PSF stretch for increasing θ (see below for details), which causes an overall drop in efficiency due to the nonlinearity of the two-photon fluorescence. The fits, shown as solid lines in Fig. 1(b), yield efficiencies η=0.71 for $\lambda_{ex}=1000$ nm and η=0.44 for $\lambda_{ex}=800$ nm. These are somewhat lower than the predicted values, possibly due to additional diffractive losses associated with the finite axial spacing of the MDOE elements, especially at 800 nm which is far from the 1064 nm design wavelength. It is worth noting that the efficiency shown here does not return to its maximum value at ${\mathrm{360}}^{\circ }$ because the two photon signal was measured only in the plane of the first MDOE focus (see Supplementary Materials for details).

Furthermore, while multiple weak foci along the optical axis are generated by the MDOE [33], the second order power dependence of the two-photon fluorescence intensity suppresses the signal from these weaker foci and no measurable two-photon signal was produced outside of the desired focal plane.

The measured axial shift Δz as a function of rotation angle is shown in Fig. 1(c) for $\lambda_{ex}=1000$ nm (red) and $\lambda_{ex}=800$ nm (blue) along with theoretical predictions (dashed lines) based on the angle-dependent spherical phase function (see Supplementary Materials for details). The power of the lens increases linearly with wavelength, leading to a measured shift of 0.456 μm/° for $\lambda_{ex}=1000$ nm compared to 0.358 µm/° for $\lambda_{ex}=800$ nm, which are both within 1% of their predicted values.

Finally, the measured first order MDOE diffraction efficiency is plotted as a function of focal shift in Fig. 1(d) with fits to the efficiency function described above shown as solid lines. While the maximum efficiencies η are the same as those found previously, this reveals that the characteristic length scale of the exponential decrease in efficiency δ=34 µm for $\lambda_{ex}=800$ nm is somewhat shorter than the δ=41 µm found for $\lambda_{ex}=1000$ nm. This arises because a larger MDOE rotation angle is required to achieve a given focal shift at 800 nm compared to 1000 nm.

### 3.2 PSF characterization

**Fig. 2.**
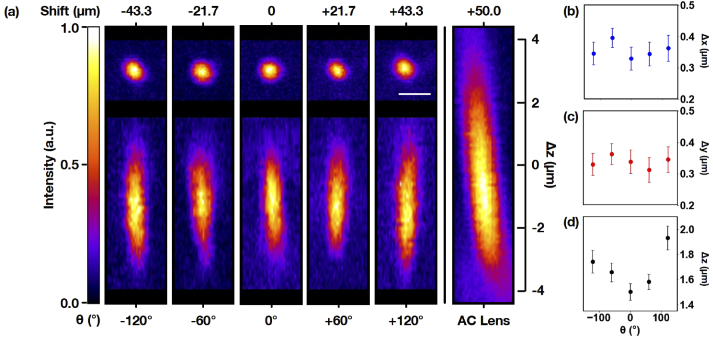
**PSF characterization.** (a) Two-photon lateral (top) and axial (bottom) imaging of the PSF shifted by the MDOE over a displacement of ± 43.3 µm corresponding to rotation angles θ up to ± 120° (left) with scale bar indicating 1 µm, and focal spot shifted by 50 µm using an achromatic (AC) lens doublet (right). (b)-(d) Fitted FWHM of the PSF in x, y, and z, respectively. All measurements were taken with an excitation wavelength of 800 nm and intensities were normalized for clarity.

A thorough investigation of the system’s resolution was performed via measurement of the optical point spread function as described in Section 2. Lateral and axial projections of PSF images covering axial shifts up to ±43.3 μm, corresponding to MDOE rotations $\theta =\pm 120^{\circ }$ along with fitted FWHM along x, y, and z axes are shown in Fig. 2. Note that the intensities of the PSF images are not directly comparable due to variation in the quantum dot brightness and have been normalized for clarity. Quantitative analysis of these images reveals that with no MDOE rotation ($\theta =0^{\circ }$), the focal spot has a width of 0.34 µm and an axial extent of 1.51 µm, corresponding to a stretch of 31% (lateral) and 37% (axial) compared to the diffraction limit. Interestingly, a larger MDOE rotation of $\theta =\pm 120^{\circ }$ leads to only a modest increase in the lateral PSF width of 5%, but causes an additional axial stretch of 30%. While some increase in the spatial extent of the PSF is likely due to aberrations caused by the imaging optics and imperfections in the MDOE generated wavefront, which is an approximation to the ideal phase valid only in a finite region around the objective’s focal plane, the additional axial stretch for $\theta =\pm 120^{\circ }$ may also partly arise due to the wavelength dependence of the MDOE focal shift. A 100 fs pulse centered at 800 nm has a spectral width of ≈7 nm or 0.9% of the central wavelength. The linearly increasing focal shift of the MDOE with wavelength means that any overall focal shift Δz imparted to the pulse will be accompanied by a 0.009 Δz dispersive broadening which manifests itself as an axial stretch of the PSF. For $\Delta \theta =120^{\circ }$, where Δz = 43.3 µm, the expected broadening is 380 nm, which is about half of the observed PSF stretch. For a full quantification and analysis of the PSF extent, please see Supplementary Materials.Finally, the PSFs formed by the MDOE are compared to that generated by placing an additional achromatic doublet (AC) lens (400 mm focal length), instead of the MDOE, into a pupil conjugated plane. This additional lens is of course not dynamic, but nevertheless introduces a well-defined wavefront curvature that is equally good or even superior to those produced by alternative remote focusing devices such as tunable polymer, electro-wetting or acousto-optic lenses. The lens may thus serve as a benchmark to judge the focus quality of the MDOE.

While the focal shift of 50 µm imparted by the additional achromat is comparable to that of the MDOE, it also introduces severe aberrations leading to an axial stretch of the PSF to over 300% of the diffraction-limited value as shown in the right panel of Fig. 2.

### 3.3 Biological imaging

**Fig. 3.**
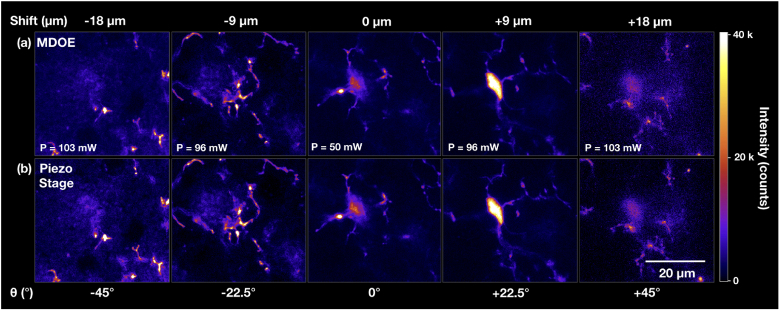
**Comparison of microglia imaging with MDOE and piezo stage.** Example images from 3D stacks taken by remote focusing with the MDOE (top) and by stepping the sample with a piezo stage to corresponding imaging depths (bottom) over focal shifts up to ±18 µm corresponding to MDOE rotation angles θ of $\pm 45^{\circ }$. Compensated laser power is shown in white for MDOE imaging and piezo stage images were acquired with P = 39 mW.

In order to assess the viability of the MDOE for axially resolved, high resolution biological microscopy, it was used to perform 3D volumetric imaging of a single microglia cell over its full extent of >40 µm with lateral and axial scan step increments of 0.05 µm and 2 µm, respectively. After each axial scan step, the excitation power was adjusted to compensate for the angle-dependent decrease in efficiency. Several representative frames from the resulting image stack are shown in Fig. 3(a) with the imaging laser power denoted in white, revealing high quality, diffraction-limited images of both the microglia soma and its processes with sub-micron feature sizes (the complete image series is shown in the Supplementary Materials). For comparison, an equivalent 3D image stack was acquired via axial translation of the sample using a piezo stage with no MDOE in the beam path. The resulting images are almost indistinguishable from those acquired using the MDOE as shown in Fig. 3(b), further validating its viability for high resolution biological imaging. Please note that the 40.6 µm imaging range covered here was limited only by image degradation due to scattering in the biologial tissue, and that for clear tissues high quality imaging with the MDOE is possible over more than double this axial range as proven by PSF measurements taken over a z-range of 86 µm (see Fig. 2).

**Fig. 4.**
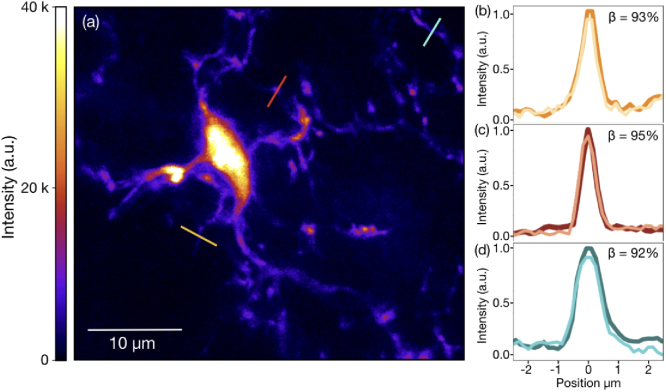
**Quantification of imaging contrast.** (a) Maximum intensity z-projection of the 3D image stack over a full microglia cell taken with MDOE lens. (b)-(d) Two photon fluorescence intensities corresponding to the locations of the gold, red, and turquoise lines, respectively, shown in (a) for images taken with axial translation performed through piezo stage stepping (dark) and MDOE rotation (light) with the relative contrast β quantified in the inset.

To further investigate the performance of the remote focusing element, maximum intensity z-projections were taken for the MDOE image stack as shown in Fig. 4(a) as well as the piezo translation stack, and were found to be nearly identical (see Supplementary Materials). The loss of imaging contrast due to undesired diffraction orders of the MDOE was then quantified by comparing the intensities of these images along the three line segments indicated in Fig. 4(a). The extracted line data are shown in Fig. 4(b)-(d) for the MDOE image (light) and the piezo translation image (dark) with colors corresponding to the lines in Fig. 4(a). The contrast ratios β between the MDOE and piezo translation images for the three regions are shown in the insets of Fig. 4(b)-(d), and indicate an average loss of contrast <7% for the MDOE lens.

## 4. Conclusion

In summary, we have demonstrated volumetric two-photon biological imaging in which single microglia cells are resolved with deep sub-micron resolution over an axial range of 40.6 µm using a novel high-NA diffractive remote focusing element for axial scanning. The spherical defocus wavefront imparted by the lens causes no measurable axial or lateral distortion to the resulting images over this range and the image contrast is maintained to within 7% compared to mechanical sample stepping, even for excitation wavelengths that are ∼25% smaller than the MDOE design wavelength. This highlights the viability of the high-NA MDOE over a broad range of visible and near-IR wavelengths [33], which is an attractive feature for resonant imaging techniques like TPEF.

Careful PSF characterization reveals that the MDOE introduces almost no aberrations to the focal spot, even over rotations up to $\pm 120^{\circ }$, corresponding to an axial translation >86 µm. In contrast, when the focus is shifted by 50 µm using an achromatic lens doublet, severe aberrations stretch the PSF to more than 300% of its diffraction-limited value.

The high-NA MDOE design used here is also suitable for experiments requiring larger axial displacements where a decreased lithographic structure size can yield focal shifts on the order of several μm${/}^{\circ }$, but pulse dispersion will then increase as well.

Finally, axial scan speeds up to several kHz could be realized for continuous rotation of the MDOE using currently available electric motors, which is nearly two orders of magnitude faster than the limit for mechanical stepping of the sample or objective [34]. This would enable resolution of even the most rapid cellular dynamics, like the firing and propagation of action potentials, without introducing high frequency vibrations.

While power loss due to the diffraction efficiency of the MDOE could pose problems in biological studies, uniform diffractive losses can generally be compensated with increased laser power. Furthermore, angle-dependent losses could be automatically compensated during axial scans by attaching an appropriate angle-dependent filter, i.e. a linear polarizer, to the rotating MDOE element.

The versatile and robust method demonstrated here is an important step toward high resolution volumetric imaging in living tissues on neurological timescales. It allows for the combination of fast 3D scanning, such as provided by acoustic scanners [35–37], with the high spatial resolution of high NA objectives. Our technique may also be combined with other strategies for improving imaging speed, such as axially scanned multiplexing in which multiple focal spots are scanned simultaneously to increase imaging speed [38–41]. Finally, in contrast to focusing by mechanically translating the focal plane, remote focusing techniques such as presented here are compatible with conjugated adaptive optics, which can correct for field-dependent aberrations [42–46].

## Notes

See Supplement 1 for supporting content.
