# Supplementary material for: High-NA two-photon single cell imaging with remote focusing using a diffractive tunable lens
Source: Biomed Opt Express. 2020 Nov 16;11(12):7183–91. doi: 10.1364/BOE.405863 (PMC7747902; doi:10.1364/BOE.405863)
Supplement: Supplementary file 1 [file boe-11-12-7183-s001.pdf]

Supplemental Document

Biomedical Optics EXPRESS

High-NA two-photon single cell imaging with remote focusing using a diffractive tunable lens: supplement

Molly A. May,[1,*] Martin Bawart,[1] Michiel Langeslag,[2] Stefan Bernet,[1] Michaela Kress,[2] Monika Ritsch-Marte,[1] and Alexander Jesacher[1]

[1] *Institute of Biomedical Physics, Medical University of Innsbruck, Müllerstraße 44, 6020 Innsbruck, Austria*
[2] *Institute of Physiology, Medical University of Innsbruck, Schöpfstraße 41, 6020 Innsbruck, Austria*
[*] *molly.may@i-med.ac.at*

This supplement published with The Optical Society on 16 November 2020 by The Authors under the terms of the Creative Commons Attribution 4.0 License in the format provided by the authors and unedited. Further distribution of this work must maintain attribution to the author(s) and the published article's title, journal citation, and DOI.

Supplement DOI: https://doi.org/10.6084/m9.figshare.13140218

Parent Article DOI: https://doi.org/10.1364/BOE.405863

High-NA two-photon single cell imaging with remote focusing using a diffractive tunable lens: supplemental document

1. THEORETICAL AXIAL SHIFT

The MDOE design presented here can be used with a wide variety of experimental parameters including different excitation wavelengths, objective lenses, and immersion media. The axial shift imparted by an MDOE as a function of rotation angle $\Delta z(\theta)$ in radians for an objective with arbitrary numerical aperture (NA), immersion medium with refractive index n, and excitation wavelength λ can be calculated using the following equation

$$\Delta z(\theta) = \frac{R\, n_d^2\, \lambda\, \theta}{\mathrm{NA}_d\, \mathrm{ss}\, n} \left(\frac{\pi^2}{n_d^2/\mathrm{NA}_d^2 - 1} + n_d^2/\mathrm{NA}_d^2 - 1 \right)^{-1/2} \tag{S1}$$

where R is the radius of the MDOE elements, n_d is the design index of refraction of the MDOE, NA_d is the design NA of the MDOE, and ss is the lithographic structure size of the diffractive elements. From this equation it is clear that the axial shift increases with wavelength, as observed in Fig. 1 (c). The lithographic structure size is a free design parameter that can be used to tune the axial shift based on the requirements of a given experiment.

2. REDUCING CROSSTALK BETWEEN DIFFRACTION ORDERS

The diffraction pattern of the MDOE is dominated by two axially separated diffraction orders, whose powers oscillate with $\mathrm{sinc}(\theta/2)^2$. During MDOE rotation, the respective stronger order takes the role of the imaging focus. The signal generated by the weaker spot can generate undesired crosstalk within a certain θ range. Placing a circular aperture in an image-conjugate plane along the excitation path causes additional suppression of undesired signal contributions from the weaker MDOE focus. The left graph in Fig. S1 shows the two-photon signals generated by both foci. The dashed curves indicate the trends when no additional aperture is used, such as in our experiments. In this case, the angular range where the weaker signal exceeds 10% of the stronger one is limited to a 100° wide interval between θ = 130° and θ = 230° (see graph on the right). An additional aperture of 2 mm diameter restricts this range to an interval of merely 60° width between θ = 150° and θ = 210°.

These data are based on our specific experimental conditions (NA = 1.0, focal length objective = 9 mm, focal length tube lens = 180 mm) and assuming a Gaussian excitation beam whose waist diameter matches the pupil diameter of the objective lens. The signals generated by the foci are calculated by integrating over the respective beam powers passing through the aperture and squaring the result. Of note, the aperture will also effect the focal spot shape, predominately broadening it by decreasing the effective NA. This effect causes a further drop in signal which is not considered in this simulation.

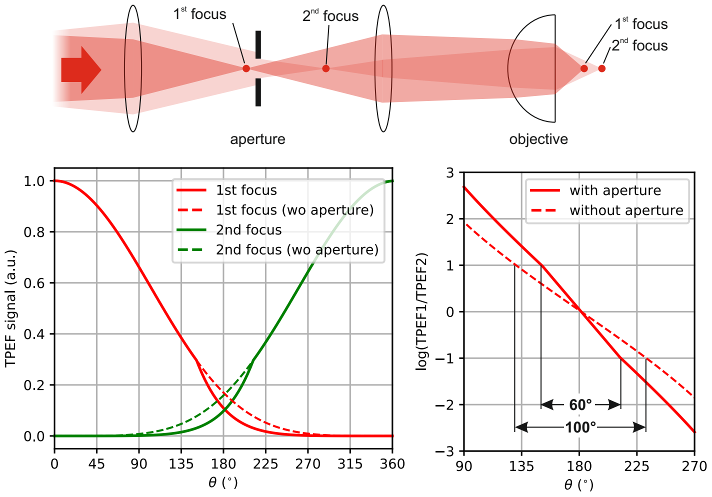

Fig. S1 Blocking undesired diffraction orders. Top: an aperture of 2 mm diameter, placed in an image-conjugate plane, leads to additional suppression of the weaker focal spot, thereby reducing crosstalk. Bottom left: two-photon signals generated by the two foci, with (solid) and without (dashed) using the additional aperture. Bottom right: Decadic log-ratio of the two-photon signals for the two scenarios.

3. FULL XYZ PSF CHARACTERIZATION

The X, Y, and Z extent of the PSFs for increasing focal shift were quantified using a 3D Gaussian fits. The resulting fitted FWHM are shown in Table S1. The full PSF imaging data sets have also been included as supplemental data.

Δz	-43.3 µm	-21.7 µm	0 µm	21.7 µm	43.3 µm
X	0.33 µm	0.36 µm	0.34 µm	0.31 µm	0.35 µm
Y	0.35 µm	0.39 µm	0.33 µm	0.353 µm	0.36 µm
Z	1.74 µm	1.66 µm	1.50 µm	1.58 µm	1.93 µm

Table S1 Fitted XYZ PSF extent. PSF extent in X, Y, and Z as determined by 3D Gaussian fits to the experimental data as a function of focal shift Δz.

4. SAMPLE PREPARATION

Mice expressing GFP under the Cx3Cr1 promotor (Cx3Cr1-GFP+/+) were bought from Jackson Laboratories and bred in-house. Animals were housed in standard pathogen free (SPF) conditions, at 24°C on a 12 h light/dark cycle and had free access to autoclaved pelleted food and water. For tissue dissection, animals were subjected to inhalation of a lethal dose of CO2. Subsequently, the spinal cord was removed and fixated with 4% PFA overnight at 4°C. After fixation, the spinal cord was placed in an agarose block to cut 300 µm transverse sections (Leica VT1000S). The spinal cord slices were then placed in a glass bottom dish (in-house made) containing phosphate buffered saline (PBS) and sealed with a second glass coverslip. All animal procedures were in accordance with ethical guidelines and animal welfare regulations according to Austrian law and with documented permission of the Austrian BMWF ministry (BMWF-66.011/0148-V/3b/2019).

5. SUPPLEMENTARY IMAGING DATA

Microglia imaging was performed over a full range of ±20.3 µm, with images taken every 2 µm. While a subset of these images is shown in Fig. 3 of the main text, the full set of images is shown in Fig. S2 for stacks taken with mechanical stepping of the sample (top) and by remote focusing with the MDOE (bottom).

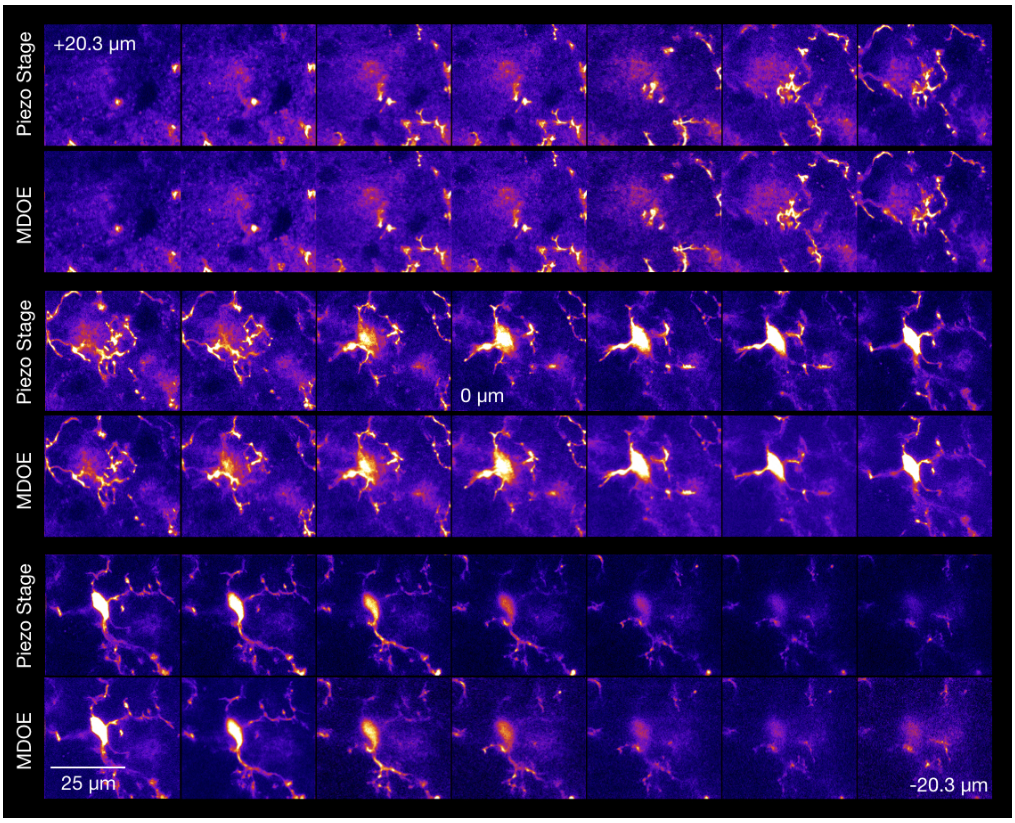

Fig. S2 Full stack of biological image sections. Two-photon images taken with 2 μm steps over the full imaging range of 40.6 μm by (a) moving the sample with a piezo stage and (b) remote focal tuning with the MDOE.

The maximum z projection of the image stack taken with the MDOE in Fig. 4 (a) is also included in Fig. S3 (a) along with with the equivalent projection for the images taken with mechanical stepping in Fig. S3 (b) for comparison.

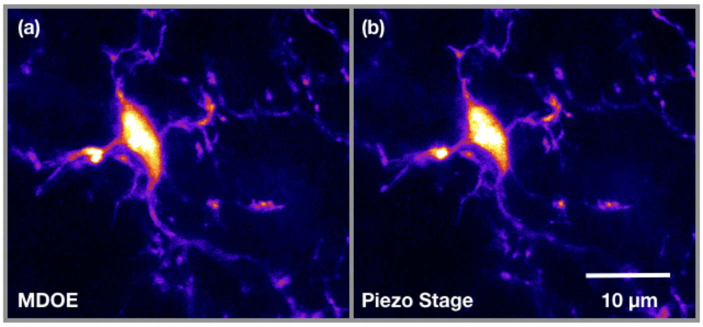

Fig. S3 Comparison of axial image projections. Maximum z-projections of image stacks taken with the MDOE (a) and piezo stage (b).
